# A Bibliometric Analysis of Atopic Dermatitis Research over the Past Three Decades and Future Perspectives

**DOI:** 10.3390/healthcare9121749

**Published:** 2021-12-17

**Authors:** Dongwon Kim, Younbyoung Chae, Hi-Joon Park, In-Seon Lee

**Affiliations:** 1 College of Korean Medicine, Kyung Hee University, Seoul 02447, Korea; dwtuna96@khu.ac.kr (D.K.); ybchae@khu.ac.kr (Y.C.); acufind@khu.ac.kr (H.-J.P.); 2 Acupuncture & Meridian Science Research Center, Kyung Hee University, Seoul 02447, Korea

**Keywords:** atopic dermatitis, bibliometric analysis, asthma, immunoglobulin E, probiotics

## Abstract

Atopic dermatitis (AD) has been increasing in prevalence over the past few decades; however, AD has never been analyzed using a bibliometric approach. We searched for AD studies in the dermatology and allergy category of the Web of Science and SCOPUS databases using the keywords “atopic dermatitis”, “eczema”, and “atopy”. In total, 53,460 documents were retrieved. We analyzed annual publication trends and performed keyword and co-authorship network analyses. The annual number of AD publications has increased over the years. Asthma, food allergies, the skin barrier, IgE, and epidemiology have received extensive attention. The keywords ‘allergic rhinitis’, ‘child(ren)’, ‘quality of life’, and ‘probiotics’ have become more commonly used in recent years. AD research has been led by only a few countries, such as the USA, Germany, and the UK, and longstanding research topics such as asthma, allergy, and the immune system continue to be important. We suggest that global collaborations, research in developing countries, and research that is more holistic (thus exploring how genes, the immune system, the environment, and the microbiome together impact AD) are necessary.

## 1. Introduction

Atopic dermatitis (AD) is a chronic skin disease characterized by severe pruritus, recurrent eczema, sleep disruption, and skin manifestations (erythema, edema, papulation, oozing and crusting, excoriation, lichenification, and dryness). AD primarily affects infants and young children, and the prevalence (15–20% in children and 1–3% in adults) and recurrence rates are high [1]. AD reduces the quality of life [2], promotes suicidal ideation [3], and imposes significant economic burdens on patients and healthcare systems [4]. Patients with AD are hypersensitive to skin irritations, certain foods, and aero-allergens. They often develop other atopic disorders such as asthma or allergic rhinitis (the “atopic march” phenomenon [5,6]). Genetic variations, an impaired skin barrier, excessive inflammation, and allergic responses involving T-helper type 2 cytokines (e.g., interleukin [IL]-4 and IL-13), immunoglobulin E (IgE), and eosinophils have been proposed to contribute to AD pathogenesis [7]. Thus, the clinical evaluation and management of AD remain challenging [8]. Recently developed drugs (dupilumab, oluminant, and rinvoq), a growing interest in patient quality of life, and new potential therapeutic targets (such as the gut and skin microbiomes [9,10,11,12,13,14]) have motivated researchers to intensify their current approaches and explore new areas of AD research.

To achieve this goal, we strongly believe that bibliometric analysis is useful for tracking the rise and fall of research topics over time and for evaluating the impacts of publications, research affiliations, and journals. A bibliometric analysis provides an overview of a large number of publications, allows for the quantitative assessment of past research, and can be used to predict future research trends [15,16,17]). Bibliometric analysis has been used to estimate research trends in various research fields, including dermatology [17]. Bibliometrics can be utilized to analyze publication trends by country and institution and to identify the main keywords and organizations associated with AD research. However, to the best of our knowledge, AD research has not been analyzed using a bibliometric approach that lacks restrictions on research subjects (see Zhong et al. 2011 [18] for a bibliometric analysis of AD treated via Traditional Chinese Medicine).

We aimed to analyze publication and citation trends by year, country, journal, and organization; to assess trends in the most frequently studied topics; and to identify keyword and organization clusters by performing network analyses. We identified the most productive countries, affiliations, and journals and the 30 top-cited publications to highlight the progress made and the landmark studies. This will guide future research on AD.

## 2. Methods

### Data Search and Analysis

We retrieved publications from the Web of Science and SCOPUS on 2 July 2021 using search topics including atopic dermatitis, eczema, and atopy. The retrieval strategies were TS = (“atopic dermatit*” OR atopy OR “atopic eczema”) for the Web of Science and TITLE-ABS-KEY (“atopic dermatit*” OR “atopic eczema” OR atopy) for SCOPUS. No date range limits were applied, but we restricted our search to articles written in English. The exclusion criteria were (1) articles not retrieved from the Web of Science or SCOPUS, (2) conference and meeting abstracts, (3) corrigendum documents, and (4) retracted publications. We merged the datasets from the Web of Science and SCOPUS and removed duplicates using the mergeDbSources function of R. All articles were evaluated by ISL.

Biblioshiny (https://www.bibliometrix.org/Biblioshiny.html, accessed on 5 July 2021) and the ‘bibliometrix’ R package [19] were used for the analysis of annual publication productivity (total number of publications per year), the total number of publications from each country and affiliation, the total number of citations for each article, and keyword frequency. The keyword frequency analysis was conducted for all three decades combined (1990–2020), and for each decade separately (1990–1999, 2000–2009, and 2010–2020), to identify trends in keywords over time.

We also performed a network analysis of keywords and a co-authorship-based network analysis of countries, using VOSviewer software (version 1.6.16; Centre for Science and Technology Studies, Leiden University of Leiden, Leiden, the Netherlands). Network analyses were conducted using a modularity-based clustering method. The parameters for analyses were as follows: unit of analysis = country or author keywords; fractional counting of keywords and affiliations; “layout attraction” = 3; and clustering resolution = 0.5. The minimum number of keyword occurrences was set to 5, and we excluded the keywords ‘atopic dermatitis’, ‘eczema’, ‘atopic eczema’, and ‘atopy’ from analysis. Countries that published more than five articles were included in co-authorship network analysis. All nodes were weighted by occurrence and the number of documents, respectively, to aid visualization. The total link strength and normalized citation of each country were explored to determine the influence of that country on AD research.

## 3. Results

### 3.1. Publication Productivity and Citations

**Figure 1:**
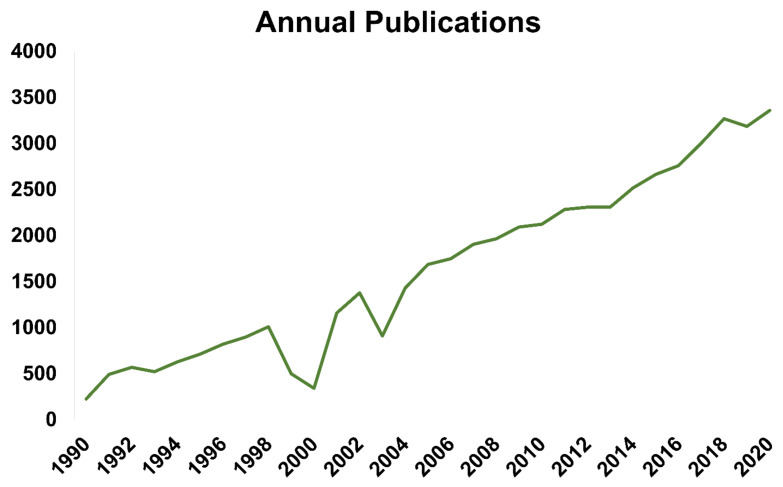
The total number of publications per year during the period 1990–2020.

A total of 53,460 articles were included after removing duplicates (*n* = 2342), conference papers/abstracts (*n* = 44), meeting abstracts (*n* = 7094), corrections, and retracted publications (*n* = 7). The annual number of publications has exhibited an upward trend since 1990 (Figure 1). The 30 most-often cited articles published between 1990 and 2020 (determined by the total citations per year) are shown in Table 1. Five studies dealt with the microbiome [20,21,22,23,24] and the skin barrier [25,26,27,28,29]; three studies explored food allergies [30,31,32]; and two studies were epidemiological in nature [33,34]. 

The 10 countries with the highest total numbers of publications were the USA, Germany, the UK, Japan, South Korea, China, France, Italy, the Netherlands, and Australia (Table 2). The total number of publications by affiliation (institution, hospital, or university) revealed that Northwestern University, the Karolinska Institute, the University of Copenhagen, the Technical University of Munich, Yonsei University, the University of California San Francisco, Seoul National University, Harvard University, the University of Munich, and the University of Helsinki were the most productive institutions; these are located in the USA, Sweden, Denmark, Germany, and Finland (Table 3).

The Journal of Allergy and Clinical Immunology, Allergy, Journal of Investigative and Clinical Dentistry, British Journal of Dermatology, Clinical & Experimental Allergy, International Journal of Advanced Biotechnology and Research, Journal of the American Academy of Child and Adolescent Psychiatry, Acta Dermato-Venereologica, Pediatric Allergy and Immunology, and Contact Dermatitis were the top 10 journals in terms of AD articles published (Table 4).

### 3.2. Keyword Frequency and Network Analyses

**Figure 2:**
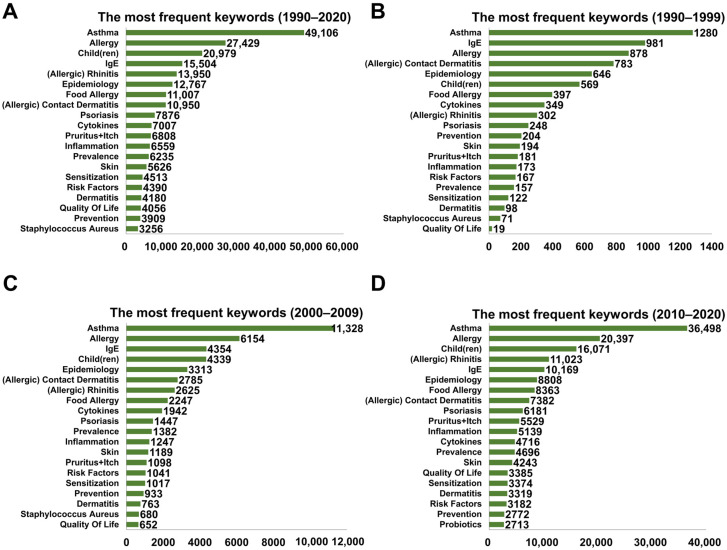
The most frequently used keywords ordered by occurrence in the period 1990–2020. (**A**) Number of the most frequently used keywords from 1990 to 2020 from publications in AD research. During the past three decades, ‘Asthma’, ‘Allergy’, ‘Child(ren)’, ‘IgE’, and ‘(Allergic) Rhinitis’ have been the most commonly used keywords; (**B**) Number of the most frequently used keywords from 1990 to 1999. During the early years of AD research, the top five most frequently used keywords were ‘Asthma’, ‘IgE’, ‘(Allergic) Contact Dermatitis’, ‘Epidemiology’, and ‘Child(ren)’; (**C**) Number of the most frequently used keywords from 2020 to 2009. The keyword ‘Child(ren)’ had become more popular than the previous decade; (**D**) Number of the most frequently used keywords from 2010 to 2020.

Keyword frequency analysis showed that the 10 most frequently used keywords were ‘asthma’, ‘allergy’, ‘child(ren)’, ‘IgE’, ‘(allergic) rhinitis’, ‘epidemiology’, ‘food allergy’, ‘(allergic) contact dermatitis’, ‘psoriasis’, ‘cytokines’, and ‘pruritus (itch)’ (Figure 2A). We also analyzed the frequency of keywords by decade as a further analysis of the changes in research trends over the past three decades. The top five most frequently used keywords between 1990 and 1999 were ‘asthma’, ‘IgE’, ‘(allergic) contact dermatitis’, ‘epidemiology’, and ‘child(ren)’ (Figure 2B). The top five most frequently used keywords between 2000 and 2009 were ‘asthma’, ‘allergy’, ‘IgE’, ‘child(ren)’, and ‘epidemiology’ (Figure 2C). The top five most frequently used keywords between 2010 and 2020 were ‘asthma’, ‘allergy’, ‘child(ren)’, ‘(allergic) rhinitis’, and ‘IgE’ (Figure 2D). These results suggest that asthma, allergy, IgE status, and childhood AD have been extensively and consistently studied over the past three decades, although the occurrence ranking of IgE decreased over that time. The ranking of the keywords ‘contact dermatitis’, ‘cytokines’, ‘IgE’, ‘prevention’, and ‘risk factor’ decreased gradually, indicating that less emphasis has been placed on those research topics by AD researchers. In contrast, the keywords ‘allergic rhinitis’, ‘child(ren)’, and ‘quality of life’ have become more popular over the decades. The keyword ‘probiotics’ entered the rankings between 2010 and 2020, revealing increasing interest in the microbiome of patients with skin disease. 

**Figure 3:**
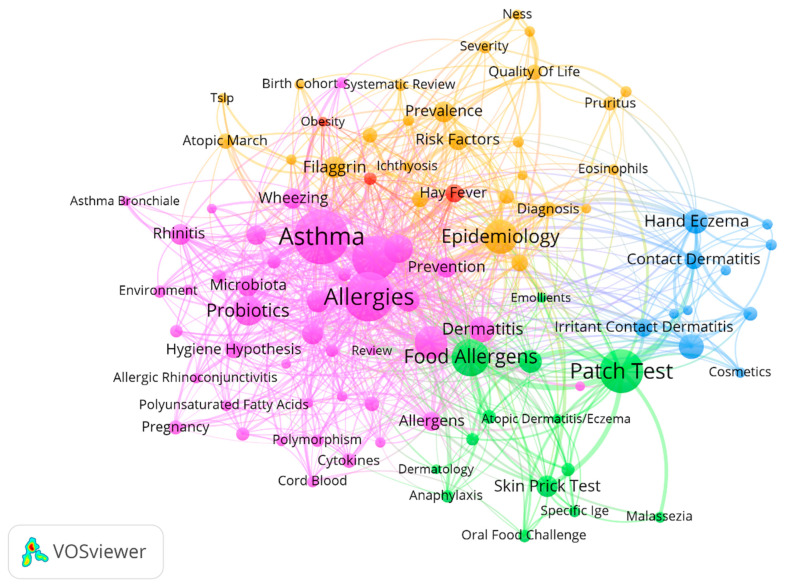
Network analysis of author keywords during the period 1990–2020. Keywords used more than five times were included in the network analysis; the keywords ‘atopic dermatitis’, ‘eczema’, ‘atopic eczema’, and ‘atopy’ were excluded. The following parameters for VOSviewer were used: layout attraction of 3, clustering resolution of 0.5, visualization scale of 1.3, and size variation of 0.6; node sizes were weighted based on the keyword frequencies in the clusters.

Of the 1800 keywords extracted from the titles and abstracts of the studies, 96 (excluding “atopic dermatitis”, “eczema”, “atopic eczema”, and “atopy”) were included in the network analysis. Five clusters were defined based on the co-occurrences of each keyword and other keywords (Figure 3 and Table 5). Cluster 1 included 42 keywords associated with asthma, allergies, and the microbiome (e.g., ‘asthma’, ‘allergies’, ‘children’, ‘IgE’, ‘microbiota’, ‘probiotics’, and ‘allergic rhinitis’). Of the keywords in Cluster 1, ‘vitamin D’ and ‘microbiota’ were relatively new (thus more commonly employed in recent years) (pink cluster in Figure 3). Cluster 2 comprised 26 keywords associated with the skin barrier and epidemiology (e.g., ‘filaggrin’, ‘prevalence’, ‘risk factors’, ‘atopic march’, and ‘quality of life’; orange cluster in Figure 3). Of the keywords in Cluster 2, ‘meta-analysis’, ‘systematic review’, ‘phenotype’, and ‘pediatrics’ were relatively new. Cluster 3 included 14 keywords associated with allergens and skin tests (e.g., ‘patch test’, ‘food allergens’, and ‘aeroallergens’; green cluster in Figure 3). Cluster 4 included 11 keywords associated with skin contact (e.g., ‘contact dermatitis’, ‘contact allergy’, ‘patch test’, ‘occupational’, and ‘wet work’; blue cluster in Figure 3). Cluster 5 included keywords associated with non-skin diseases (‘hay fever’, ‘obesity’, and ‘rhinoconjunctivitis’; red cluster in Figure 3).

### 3.3. Co-Authorship Network Analysis

**Figure 4:**
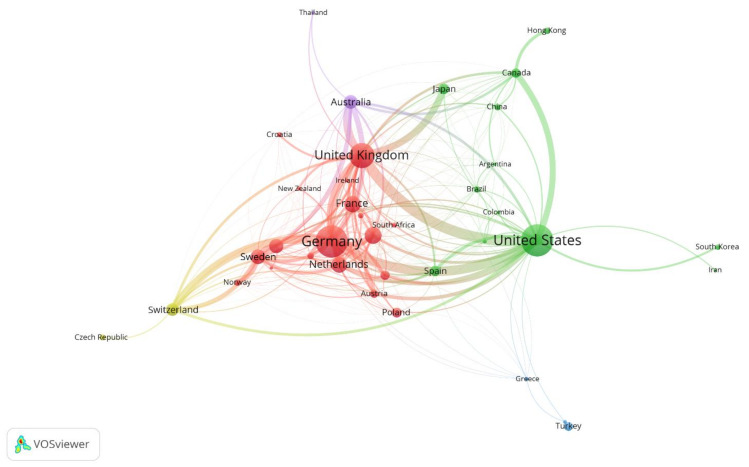
Co-authorship networks of countries during the period 1990–2020. Organizations that published more than five articles were included in the co-authorship network analysis. The following parameters for VOSviewer were used: layout attraction of 3, clustering resolution of 0.5, visualization scale of 1.3, and size variation of 0.6; the node sizes were weighted based on the number of documents. Five clusters were identified. The USA, the UK, and Germany were central hubs of collaborative research.

Of the 103 countries, 38 were included in the co-authorship analysis, and four clusters were identified (Figure 4 and Table 6). Cluster 1 included 18 countries, almost all of which are European (e.g., Germany, the UK, Italy, France, and the Netherlands; red cluster in Figure 4). Co-authorship connections were also found between European countries and Singapore, Austria, or New Zealand (Cluster 1). Cluster 2 contained 12 countries in the Americas (including the USA, Brazil, and Mexico), Canada, and East Asia (Japan, China, Hong Kong, and South Korea; green cluster in Figure 4). Cluster 3 included Turkey, Greece, and India (blue cluster in Figure 4). Cluster 4 included Switzerland and the Czech Republic (yellow cluster in Figure 4). Cluster 5 included Australia and Thailand (purple cluster in Figure 4). 

Germany, the USA, the UK, Sweden, and the Netherlands exhibited the greatest normalized citation scores (326, 311, 249, 151, and 144, respectively) and total link strengths (in a slightly different order), suggesting that these countries exert the most influence on AD research in terms of both impactful papers and global collaborations that have been in operation for decades. 

## 4. Discussion

Analysis of the 53,460 articles collected from the Web of Science and SCOPUS databases showed that the number of publications on AD has increased since 1990. The citation and keyword frequency analyses revealed that research topics such as asthma, food allergies, the skin barrier, IgE, and epidemiology have received extensive attention. The rankings of the keywords ‘contact dermatitis’, ‘cytokines’, ‘IgE’, ‘prevention’, and ‘risk factor’ decreased gradually over time, while the keywords ‘allergic rhinitis’, ‘child(ren)’, ‘quality of life’, and ‘probiotics’ have become more popular. We identified five main AD-related topics (inflammation and allergy, the skin barrier, allergens, contact dermatitis, and non-skin diseases). The USA, Germany, the UK, Japan, and South Korea were the top five most productive countries in the field of AD research. The USA, Germany, the UK, Sweden, and the Netherlands exerted the greatest influence on AD research. 

The most cited article (published in 2006) addressed the prevalence of eczema in childhood [33]. That study conducted a cross-sectional questionnaire survey in 193,404 children from 37 countries (aged 6–7 years) and 304,679 children from 56 countries (aged 13–14 years). The top citation rate suggests that researchers are very interested in the different prevalences of eczema worldwide; geographical variation may be important in terms of the development of asthma and allergies. The cited authors found that the prevalences of eczema symptoms in both groups increased over time in most regions but decreased in some. An understanding of the factors promoting the decreases might allow the effective prevention and treatment of allergic diseases. The fourth most-cited article dealt with the skin microbiome [20], suggesting that microbial factors may contribute to AD development. Indeed, recent studies have suggested that an impaired skin microbiome can affect both the skin barrier [12] and the immune environment [14], eventually causing AD development and worsening the symptoms [13].

### 4.1. Sustainable and Newly Highlighted Research Themes

AD is frequently associated with respiratory allergic diseases, such as asthma and allergic rhinitis. The term “atopic march”, which refers to the progression of symptoms from AD to asthma to allergic rhinitis during childhood, emphasizes the strong association between AD and respiratory allergy. While the preventative effects of treating atopic march are controversial, IgE-associated allergic reactions are regarded as a key mechanism of AD, and our keyword frequency analysis emphasized the significance of the keywords ‘asthma’, ‘allergy’, and ‘IgE’ (the ranking of the latter decreased over time, but it still remains in the most recent top five). As AD is a chronic and recurrent disease, managing quality of life has become a major concern when treating AD patients. In contrast, the keywords ‘contact dermatitis’ and ‘cytokine’ dropped in the rankings, which might reflect the reduced interest of AD researchers in contact dermatitis. However, as AD patients may have an increased risk of contact sensitization, and contact dermatitis is more severe in patients with than without AD [35,36], studies that focus on the weak, sensitized skin of AD patients remain meritorious. We performed citation and keyword frequency analyses to identify topics that have recently received significant attention. These topics included the microbiome and probiotics. The roles played by the microbiological environments of the skin and other organs (e.g., the gut) remain poorly understood, as do the effects of probiotics [9]. As patients are now increasingly exposed to commercial probiotics, any abnormal changes in the composition or functions of the AD microbiome require attention, as do any effect of probiotics on AD. Such work might yield new treatments for AD. 

### 4.2. Insufficient Global Collaborative Research Network 

The co-authorship network analysis identified five clusters of countries. The UK, Germany, the USA, the Netherlands, and Sweden were influential research hubs; these countries scored highest in terms of total link strength. On the other hand, Japan and South Korea were both very productive, but the citation scores were rather low, suggesting that research quality requires improvement. The clusters consisted mainly of neighboring countries or those on the same continent, indicating that there are only a few intercontinental global collaborations in AD research. Building an international network to strengthen the research community, and enhance research quality and diversity, could be valuable. 

### 4.3. Future Directions

It is important to note that although AD is a worldwide disease, research thereon has mostly been performed in a few developed countries and may not be transferable to AD patients in developing countries. Thus, AD patients in developing countries might not benefit from the research findings, even though studies have shown an increasing prevalence of AD in such countries [33,37]. For example, the mechanisms and risk factors in developing countries could be different to those in developed countries. Therefore, it is necessary to train healthcare providers and improve patient access to healthcare resources, as well as to facilitate AD research, in developing countries. 

In addition to skin barrier impairments in the context of food and skin contact, AD is also related to environmental factors (e.g., climate, UV light exposure, temperature, and air pollution), diet, and the gut and skin microbiomes [38]. We suggest that collaborations among epidemiologists, basic scientists, clinical researchers, microbiologists, and pharmaceutical industrialists, and the establishment of international research networks, will improve our knowledge of the causes of AD and enable us to treat AD more effectively in the future. 

### 4.4. Limitations

This study had several limitations. The quality of the publications was not considered, and all authors were treated equally regardless of their contribution to the work (e.g., first, corresponding, or co-author). Finally, some institutes might have close relationships (e.g., the Karolinska Institute and Karolinska University Hospital), although we changed or combined organization names to account for this. 

## 5. Conclusions

In conclusion, the countries leading AD research have been the USA, Germany, the UK, Japan, South Korea, Sweden, and the Netherlands. The most prevalent research themes have been ‘asthma’, ‘allergy’, ‘child(ren)’, ‘IgE’, ‘(allergic) rhinitis’, ‘epidemiology’, ‘food allergy’, ‘(allergic) contact dermatitis’, ‘psoriasis’, ‘cytokines’, and ‘pruritus (itch)’. Five research topic clusters were identified (inflammation and allergy, skin barrier, allergens, contact dermatitis, and non-skin diseases). Our analyses identified global temporal trends and the current status of AD research and suggest possible future research hotspots. For example, the lack of international research networks and interdisciplinary studies, and the few publications from developing countries, are limitations of AD research. We suggest that global collaborations, research in developing countries, and research on multiple factors (genes, the immune system, the environment, and the microbiome) are required in the field of AD research. 

## Figures and Tables

**Table 1:** The top 30 most cited papers on atopic dermatitis over the past 30 years.

No.	Articles	Total Citation/Year	Topic
1	Worldwide time trends in the prevalence of symptoms of asthma, allergic rhinoconjunctivitis, and eczema in childhood: ISAAC Phases One and Three repeat multicountry cross-sectional surveys	161.56	AD epidemiology
2	Randomized Trial of Peanut Consumption in Infants at Risk for Peanut Allergy	134.29	Food allergy
3	Global epidemiology of psoriasis: a systematic review of incidence and prevalence	132.89	Other disease (psoriasis)
4	The skin microbiome	118.82	Microbiome
5	Two Phase 3 Trials of Dupilumab versus Placebo in Atopic Dermatitis	117.5	Clinical trial
6	Unbiased classification of sensory neuron types by large-scale single-cell RNA sequencing	114.86	Itch
7	Common loss-of-function variants of the epidermal barrier protein filaggrin are a major predisposing factor for atopic dermatitis	114.19	Skin barrier
8	Guidelines for the diagnosis and management of food allergy in the United States: report of the NIAID-sponsored expert panel	112.33	Food allergy
9	Worldwide variation in prevalence of symptoms of asthma, allergic rhinoconjunctivitis, and atopic eczema: ISAAC	111	AD epidemiology
10	Dermatology Life Quality Index (DLQI)—a simple practical measure for routine clinical use	98.75	Questionnaire
11	Prebiotic effects: metabolic and health benefits	93.67	Microbiome
12	Atopic Dermatitis	91	Review
13	International Study of Asthma and Allergies in Childhood (ISAAC): rationale and methods	88.70	Study introduction
14	The effect of infections on susceptibility to autoimmune and allergic diseases	85.65	Infection
15	Temporal shifts in the skin microbiome associated with disease flares and treatment in children with atopic dermatitis	81.2	Microbiome
16	Exposure to Environmental Microorganisms and Childhood Asthma	81.09	Microbiome
17	Revised nomenclature for allergy for global use: Report of the Nomenclature Review Committee of the World Allergy Organization, October 2003	79.89	Nomenclature
18	Probiotics in primary prevention of atopic disease: a randomised placebo-controlled trial	79.76	Microbiome
19	The development of allergic inflammation	75.07	Review
20	Human epithelial cells trigger dendritic cell–mediated allergic inflammation by producing TSLP	70.75	Skin barrier
21	Eosinophilic esophagitis in children and adults: a systematic review and consensus recommendations for diagnosis and treatment	69.53	Other disease (eosinophilic esophagitis)
22	Pathogenesis and clinical features of psoriasis	69.07	Other disease (psoriasis)
23	Endogenous Antimicrobial Peptides and Skin Infections in Atopic Dermatitis	66.15	Skin barrier
24	The skin: an indispensable barrier	62.93	Skin barrier
25	A revised nomenclature for allergy. An EAACI position statement from the EAACI nomenclature task force	58.71	Nomenclature
26	Impaired TH17 cell differentiation in subjects with autosomal dominant hyper-IgE syndrome	58.21	Other disease (hyper-IgE syndrome)
27	IL-22 increases the innate immunity of tissues	56.56	Interleukin
28	New insights into atopic dermatitis	55.89	Review
29	Skin immune sentinels in health and disease	55.31	Skin barrier
30	Update on food allergy	54.28	Food allergy

**Table 2:** The total number of publications from the top 30 countries during the period 1990–2020.

No.	Country	Total Publications
1	USA	31,851
2	Germany	15,071
3	UK	11,611
4	Japan	11,500
5	South Korea	9810
6	China	8460
7	France	7343
8	Italy	6314
9	Netherlands	5604
10	Australia	5162
11	Sweden	4859
12	Spain	4116
13	Canada	4071
14	Denmark	3837
15	Finland	3033
16	Switzerland	2815
17	Turkey	2651
18	Poland	2414
19	Brazil	2031
20	Austria	1468
21	Iran	1424
22	India	1374
23	Norway	1280
24	Belgium	1271
25	Singapore	1130
26	Israel	1084
27	Ireland	955
28	New Zealand	952
29	Greece	764
30	Portugal	757

**Table 3:** The total number of publications from the top 30 affiliations during the period 1990–2020.

No.	Affiliation (Country)	Total Publications
1	Northwestern Univ. (USA)	1371
2	Karolinska Inst. (Sweden)	1285
3	Univ. Copenhagen (Denmark)	1026
4	Tech. Univ. Munich (Germany)	819
5	Yonsei Univ. (South Korea)	776
6	Univ. California San Francisco (USA)	772
7	Seoul Natl. Univ. (South Korea)	756
8	Harvard Univ. (USA)	724
9	Univ. Munich (Germany)	710
10	Univ. Helsinki (Finland)	675
11	Johns Hopkins Univ. (USA)	630
12	Univ. Pennsylvania (USA)	627
13	Icahn Sch. of Med. at Mountain Sinai (USA)	624
14	Univ. Southampton (UK)	588
15	Univ. Groningen (Netherlands)	585
16	King’s college London (UK)	582
17	Univ. California San Diego (USA)	580
18	Hannover Med. Sch. (Germany)	568
19	Kyung Hee Uni. (South Korea)	568
20	Chinese Univ. Hong Kong (Hong Kong)	567
21	Univ. Melbourne (Australia)	566
22	Univ. Western Australia (Australia)	551
23	Univ. Amsterdam (Netherlands)	546
24	Oregon Health and Science Univ. (USA)	535
25	Univ. Nottingham (UK)	505
26	Univ. Colorado (USA)	495
27	Univ. Toronto (Canada)	482
28	Univ. Manchester (UK)	473
29	Univ. Med. Center Utrecht (Netherlands)	440
30	Univ. Utrecht (Netherlands)	437

**Table 4:** The top 30 journals in terms of atopic dermatitis publications over the past 30 years.

No.	Journal	Total Publications	No.	Journal	Total Publications
1	Journal of Allergy and Clinical Immunology	3269	16	Current Opinion in Allergy and Clinical Immunology	469
2	Allergy	2528	17	Revue Française d’Allergologie	466
3	Journal of Investigative and Clinical Dentistry	2395	18	Archives of Dermatological Research	462
4	British Journal of Dermatology	2105	19	Der Hautarzt	429
5	Clinical & Experimental Allergy	1878	20	PLOS One	425
6	International Journal of Advanced Biotechnology and Research	1314	21	Veterinary Dermatology	418
7	Journal of the American Academy of Child and Adolescent Psychiatry	1182	22	Pediatric Dermatology	415
8	Acta Dermato-Venereologica	1046	23	Allergologie	400
9	Pediatric Allergy and Immunology	999	24	American Journal of Respiratory and Critical Care Medicine	399
10	Contact Dermatitis	969	25	The Journal of Dermatology	393
11	Annals of Allergy, Asthma and Immunology	772	26	European Respiratory Journal	355
12	International Archives of Allergy and Immunology	686	27	Journal der Deutschen Dermatologischen Gesellschaft	339
13	Journal of the European Academy of Dermatology and Venereology	673	28	Dermatology	324
14	Journal of Dermatological Case Reports	660	29	Annales de Dermatologie et de Vénéréologie	319
15	Experimental Dermatology	655	30	Journal of Immunology	314

**Table 5:** Top 10 keywords in each cluster based on keyword network analysis (sorted by total link strength).

Cluster	Keywords	Total Link Strength	Occurrences	Average Publication Year
Cluster 1	Asthma	142	154	2010
	Allergies	114	124	2010
	Children	91	100	2009
	IgE	47	54	2008
	Probiotics	40	47	2010
	Allergic Rhinitis	39	42	2012
	Dermatitis	29	34	2011
	Sensitization	26	27	2009
	Rhinitis	22	23	2011
	Allergic Diseases	22	25	2010
Cluster 2	Epidemiology	51	60	2006
	Filaggrin	22	24	2012
	Prevalence	19	23	2009
	Risk Factors	17	21	2009
	Skin Barrier	13	13	2011
	Treatment	13	15	2010
	Meta-Analysis	11	11	2015
	Quality of Life	11	12	2013
	Diagnosis	11	11	2005
	Severity	8	8	2011
Cluster 3	Patch Test	75	97	2006
	Food Allergens	66	73	2010
	Aeroallergens	26	27	2006
	Skin Prick Test	22	24	2006
	Urticaria	8	10	2006
	Oral Food Challenge	8	8	2009
	Cow’s Milk Allergy	7	9	2007
	Anaphylaxis	7	8	2009
	Food	7	8	2009
	Malassezia	6	7	2005
Cluster 4	Allergic Contact Dermatitis	28	32	2010
	Contact Allergy	4	7	2007
	Contact Dermatitis	14	18	2012
	Contact Urticaria	2	6	2000
	Cosmetics	6	6	2017
	Hand Eczema	27	31	2005
	Immunology	3	5	2007
	Irritant Contact Dermatitis	17	17	2011
	Occupational	10	10	2002
	Therapy	3	5	2008
Cluster 5	Hay Fever	17	17	2007
	Obesity	4	5	2013
	Rhinoconjunctivitis	8	8	2011

**Table 6:** Top 10 countries in each cluster based on co-authorship network analysis during the period 1990–2020 (sorted by total link strength).

Cluster	Countries	Total Link Strength	Normalized Citation
Cluster 1	United Kingdom	68	248.76
	Germany	65	326.07
	Netherlands	34	143.96
	Sweden	33	150.96
	Italy	28	110.06
	Austria	16	31.75
	Finland	15	141.94
	France	14	73.85
	Denmark	14	98.86
	Belgium	12	54.05
Cluster 2	United States	64	311.38
	Canada	19	46.66
	Spain	13	41.09
	Japan	10	48.08
	Brazil	7	19.07
	Mexico	6	33.99
	China	5	17.47
	Argentina	5	8.10
	Colombia	3	5.07
	Hong Kong	3	12.95
Cluster 3	Greece	3	15.51
	India	2	2.27
	Turkey	1	12.69
Cluster 4	Switzerland	25	85.53
	Czech Republic	1	2.92
Cluster 5	Australia	30	114.33
	Thailand	2	2.62

## Data Availability

All data are available upon request to the corresponding author.
